# Teaching Undergraduate Students to Visualize and Communicate Public Health Data with Infographics

**DOI:** 10.3389/fpubh.2017.00315

**Published:** 2017-11-24

**Authors:** Justin D. Shanks, Betty Izumi, Christina Sun, Allea Martin, Carmen Byker Shanks

**Affiliations:** ^1^MSU Library, Montana State University, Bozeman, MT, United States; ^2^OHSU-PSU School of Public Health, Portland State University, Portland, OR, United States; ^3^Community Health Program, Portland State University, Portland, OR, United States; ^4^Food and Health Lab, Montana State University, Bozeman, MT, United States

**Keywords:** infographics, health education, pedagogy, undergraduate education, communicating science

## Abstract

The purpose of this study was to explore the degree to which an infographic assignment facilitated student learning around health science issues, as well as the ways in which the assignment was an effective teaching tool. The objectives of the assignment were to (1) understand the purposes of and potential uses for infographics, (2) cultivate creative visual communication skills, and (3) disseminate a complex health topic to diverse audiences. The infographic assignment was developed at Montana State University and piloted at Portland State University. Students were assigned to small groups of three or four to create an infographic focused on a health science issue. The assignment was divided into four steps: brainstorming, developing, designing, and finalizing. Focus groups were conducted to assess how learning occurred throughout the assignment and identify any opportunities for modification of the assignment. This study was conducted with freshman students enrolled at Portland State University, a public university located in downtown Portland, OR, USA. Thirty four students completed the assignment and 31 students participated in one of three focus groups. Four themes emerged from focus groups: (1) Communicating Science-Related Topics to Non-experts, (2) Developing Professional Skills, (3) Understanding Health Issues, and (4) Overall Experience. This article outlines the assignment, discusses focus group results, and presents assignment modifications. It is clear that the infographic assignment facilitated learning about accessing and translating data. This assignment is ideally suited for use with diverse college-age audiences in health education and health promotion fields.

## Background and Rationale: Digital Technology and Higher Education Pedagogy in the Health Sciences

From static chalkboards to collaborative Internet, the contemporary higher education environment contains multitudes of tools to facilitate teaching and learning. Educational technology research shows the changing nature of students’ access to information and engagement in deep learning. Research about digital technology in higher education indicates that students today utilize social media to research information that they already perceive as credible (1) and successfully learn course content through social media platforms that extend the classroom (2).

Digital technologies undoubtedly influence how learners learn (3). Digital technologies create opportunities to open new doors to information, facilitate creativity, and inspire deeper learning (4). With growing popularity of digital technologies in higher education, it is critically important to consider how digital technologies reorient the purpose and practice of teaching. To successfully engage students in this technology-rich learning environment, faculty must carefully construct assignments around each technology and provide ongoing support (5).

In recent years, infographics have become a popular digital technology for sharing information graphically in various sectors, including the news media, business, social media, and research (6). Despite the growing body of research about digital technology use in higher education, there is little scholarship specifically addressing the pedagogical benefits of infographics, particularly in the health sciences field. This paper seeks to fill that gap by demonstrating how infographics can function as a creative assignment within the health sciences that promotes deep learning, requires critical information analysis, and facilitates collaboration.

## Pedagogical Framework and Principles: Infographics for Critical Teaching and Learning

Infographics are graphical depictions of complex information that rely upon visual elements to clearly and concisely communicate data, concepts, and/or ideas to diverse audiences (7). Infographics use evidence- and practice-based data, compelling statistics, easy-to-read fonts, complimentary color schemes, simple charts, bold graphs, and other graphics to disseminate information in quick and easily digestible format.

Infographics hold potential to serve as a critical teaching and learning tool that effectively combines student technology skills (existing or emergent) and disciplinary domain knowledge. “Similar to a traditional research essay, an infographic assignment challenges students to visually communicate a thesis, supported by citations and statistics sourced from the scholarly literature…” (8). However, infographics are more than a substitute for the traditional research essay. As a teaching tool, infographics also allow students to engage with three transdisciplinary topics important to health sciences education: (1) information literacy, (2) communicating science, and (3) data visualization.

### Information Literacy

The health sciences field requires skills to locate and vet data. Infographics are increasingly used to present data that are highly accurate and easily digestible, thereby bolstering the health literacy of end-users (9). Requiring discovery and appraisal of information available in valid and reliable channels, infographics are an important exercise for the health sciences students.

### Communicating Science

After locating and vetting data, students in the health sciences field need to develop the ability to communicate scientific ideas to their peers and the public for use in professional practice. Traditional mediums for communicating scientific information (e.g., manuscripts, books, conferences) are valuable, but often inaccessible to non-scientists. Infographics provide a mode of rapid and concise communication through visuals and have become an increasingly common method for disseminating health research to audiences in need of that health information (10, 11).

### Data Visualization

In addition to discovery, vetting, and translation of data, infographics also require the developer to think strategically about how data are visualized. Effective visualization of data requires the infographic author to utilize methods that enable the infographic reader to quickly grasp complex concepts (12). Data visualization has become so widely used in the health sciences field that the National Institutes of Health publishes various tools and techniques to visualize data (13).

## Methods

### Infographic Assignment

The infographic assignment was developed and pilot tested in summer 2016 by authors JDS and CBS. This paper focuses on further testing of the revised assignment in fall 2016 by authors BI, CS, and AM with technical assistance from authors JDS and CBS.

Students were assigned to small groups of three or four to create an infographic focused on a health issue. The objectives of the assignment were to (1) understand the purposes of and potential uses for infographics, (2) cultivate creative visual communication skills, and (3) disseminate information about a complex health topic to diverse audiences. The assignment was divided into four steps: (1) brainstorming, (2) developing, (3) designing, and (4) finalizing. In the brainstorming step, students familiarized themselves with the infographic genre, selected a topic, used research skills to identify professional sources of information about their topic, and began drafting their infographic. In the developing step, students used Piktochart (or other software) to develop their infographic. In the designing step, they selected fonts and colors for their infographic. As part of the finalizing step, students market tested their infographic with friends, family, peers, and faculty.

### Setting and Participants

This study was conducted during fall term 2016 with predominantly freshman students enrolled in one section of Freshman Inquiry (FRINQ) at Portland State University, a public university located in downtown Portland, OR, USA. Portland State University serves a racially and ethnically diverse study body and of the approximately 21,000 undergraduate students enrolled during the 2016–2017 academic year, 38% were part-time students and more than 70% received financial aid. FRINQ is a year-long, 15 credit, theme-based sequence designed to introduce freshman students to Portland State University, general education program. During each 10-week term, FRINQ students meet two times per week in faculty-led main sessions and two times per week in small group sessions led by a trained upper division student (i.e., mentor sessions).

The current study was conducted with FRINQ students enrolled in one section of the FRINQ theme, Health, Happiness, and Human Rights. Students enrolled in this FRINQ section had a diverse set of majors. The most popular major was health-related (e.g., health studies, applied health and fitness; *n* = 11), followed by undeclared (*n* = 5). Other students majored in biology (*n* = 3), psychology (*n* = 3), social work (*n* = 3), business administration (*n* = 2), political science (*n* = 1), art (*n* = 1), communication (*n* = 1), and computer science (*n* = 1).

Students worked in small groups throughout the term to complete the infographic assignment. The infographic assignment had two components: (1) creation of the infographic and (2) 10-min in-class presentation of the infographic. The infographic assignment was worth 40% of the course grade.

### Focus Groups

After completion of the infographic assignment, students were recruited to participate in a focus group held during their mentor sessions. Of the 34 students enrolled in the course, 31 participated in one of the three focus groups. The focus groups were facilitated by a researcher AM who was not involved in the class. At the beginning of each focus group, the facilitator described the purpose of the focus group and reminded students that their comments were confidential. Each focus group lasted approximately 40 min. The focus groups were semi-structured with questions (see Table 1) that focused on the students’ experience with the infographic assignment.

**Table 1:** Semi-structured focus group questions about infographic assignment.

How would describe your overall experience with this project? What was the easiest part of developing your infographic? What resources, tools, or support were helpful to you in developing your infographic? What was the hardest part of developing the infographic? What challenges did you experience when developing your infographic? How did you group overcome these challenges? Did this project help you increase your understanding of health? [If yes] Which aspects of this project were useful in helping you to better understand course material? Would you recommend this assignment for future students in this course? Why or why not? What advice would you give to future students about developing an infographic for this course? How would you recommend that a group effectively and efficiently accomplishes an infographic assignment together? What else would you like to share about the project or your experience with the project?

The focus groups were audio-recorded and transcribed verbatim by the focus group facilitator. The data were analyzed using applied thematic analysis (14). A codebook was created by one of the course instructors with 4 themes and 12 operationally defined codes based on the focus group guide and preliminary review of the transcripts. Two researchers BI and CBS independently coded all three focus group transcripts and compared them for interrater reliability. Coding discrepancies were resolved through discussion until 100% agreement was reached. After all of the focus groups were coded, all authors met to discuss themes and subthemes across the focus groups and to draw and verify conclusions about the data.

The Institutional Review Board at Portland State University approved this study.

## Results

Four themes and 12 subthemes emerged from the data (Table 2). Subthemes are organized thematically.

**Table 2:** Student focus group quotes about infographic assignment.

Theme	Subtheme	Select student quotes
Communicating science-related topics to non-experts	Translation	“[Infographics] helped show how to put a lot of information into a really short, concise statement, and make it very easy for people who don’t necessarily read medical jargon… to understand.”
	Visual representation	“One of the challenges was trying not to add too much information because [the infographic is] mostly visual. So sometimes you’re used to explaining things a lot, but on the infographic you can’t really do that, it’s just the main points.”
	Market-testing	“[Infographics] helped everyone to look at practicing new skills. For example, the market testing that we did…helped me contact other professors, other people that I don’t talk to face to face but through the internet, and try to ask for feedback for the infographic.”
Developing professional skills	Infographic	“I remember I’d seen infographics before but…there’s actually a process to this whole thing, it’s not just a bunch or random facts put on a piece of paper.”
	Professional articles	“[Reading professional articles] was cool; you’re reading what actual medical professionals read and stuff. It got me, at least, more used to understanding their lingo.”
	Resources	“We’d reserve a [study] room that just had a white board so we wrote out planning our presentation, and the last time we met we got a projector room and we were practicing our presentation in there so that was interesting to actually do that. I think that’s what I took away from [the infographic assignment] the most, actually having to go in and reserve a study room.”
	Team work	“[The infographic assignment] helped me communicate more with my peers as as partners, and helped me manage my time a lot too.”
Understanding health issues	Understanding	“[The infographic assignment] kind of opens your mind. You’re focused this whole time on your specific health disparity, but then you realize there’s a lot of other ones out there that we should be concerned about.”
	Depth	“I feel like this was an opportunity for us to get a little more in depth and find out about topics that aren’t really talked about.”
	Breadth	“There were a lot of interesting health topics covered that every group covered with their infographics, and that was actually pretty interesting. It was pretty interesting to learn what they researched, and it was nice to learn from those students, from their hard work.”
Overall experience	Positive	“I liked how [the infographic assignment] mixes research and artistic stuff and design, and [you] also learn how to put stuff together in a short thing that people can understand. It’s a good project, in my opinion.”
	Negative	“I kinda want to be thrown into [the infographic assignment] a little more. I mean, I’m lazy, I like to be lazy, but at the same time, this is something cool. This is a cool design project. And I wish instead of being handed something, we were thrown out there a little bit more.”

### Communicating Science-Related Topics to Non-Experts

Focus group participants discussed that the infographic assignment taught key skills in learning how to communicate science-related topics to non-experts through three subthemes: Translation, Visual Representation, and Market-testing. Students found that the infographic assignment facilitated practice translating science-related topics to the general public. Students learned how to visually represent science information to non-experts due to the constrained space that forces the author to distil information in an easily digestible format. Market-testing was a requirement of the assignment to understand which pieces of the infographic were effectively or ineffectively communicated. Students noted the importance of the market-testing step in refining their infographic.

### Developing Professional Skills

The infographic assignment facilitated development of professional skills through four subthemes: Infographic, Professional Articles, Resources, and Team Work. First, students learned the important skill of creating an infographic. Through the process of creating an infographic, students learned how to access information resources (such as the library’s resources) and to identify, read, and summarize professional articles, including development of an annotated bibliography. For some students, accessing information resources and using professional articles were new skills, while others discussed learning these particular skills in high school. Since students completed the assignment in teams, professional skills around team work were discussed in depth. Skills around communication, time management, and meeting deadlines were positive aspects of teamwork. Some participants discussed that it was difficult to find time to meet and to learn about the health topic or assignment in depth because they had delegated a small piece of the assignment to each team member.

### Understanding Health Issues

Focus group participants discussed the ways in which the infographic assignment did or did not facilitate learning about health issues through three subthemes: Understanding, Depth, and Breadth. It was clear that the infographic assignment facilitated student learning around health issues. Students discussed that the infographic assignment promoted deep learning about one health topic and that the in-class presentations helped students learn a breadth of health issues. Some students commented that working in a team decreased the amount of information that each individual learns about a health issue because of the shared responsibilities in the assignment.

### Overall Experience

The infographic assignment was received by students as a positive experience. Students recommended the assignment for other courses in the future, with a few adjustments. Students came to the project with differing levels of experience. Students with more advanced skills provided negative feedback that the project was elementary or drawn out over too long a timespan, while other students with less developed skills commented that the assignment matched their abilities. Students were provided with sample infographic software to use, but provided with latitude to research other available software if desired. Most students selected to use the sample infographic software and some commented that it was not user friendly.

## Discussion: Recommendations for Assignment Framework

The assignment was honed based on student feedback and focus group findings. The assignment was reframed in the following ways:

- Develop a baseline skills assessment for students to complete about key skills needed in infographic development, including knowledge of infographics, infographic use, technology use, experience locating and evaluating professional articles, accessing resources that will be useful for infographic development (e.g., library, study rooms), teamwork experiences, and presentation skills. This baseline assessment can be used to tailor preliminary classroom activities before students begin the infographic assignment.
- Encourage student to search for and experiment with infographic software at the onset of the assignment. This will empower students to select the software that best suits their technological skills and creative needs.
- Provide guidance on data visualization, potentially with a university librarian or other data visualization expert. A data visualization session should focus specifically on graphic design, choosing images, locating stock images, and providing examples from the health sciences field.
- The infographic assignment can be successfully completed either by a team or an individual. Provide an option, if possible. At the same time, think about the amount of time required for in-class presentations, which can vary greatly depending upon the number of final infographics.
- If teamwork is going to be a requirement for completion of the assignment, the authors recommend teamwork activities that teach about working with various personalities and work styles and build skills for collaboration.
- Researching, evaluating, and translating credible resources is a key component of infographic creation. Many times, students rely more heavily upon Internet search queries rather than library databases (15). As such, it is critical that students have basic research skills and know how to identify professional articles, format references, and understand ethics associated with using other people’s work.
- Think carefully about timing of the infographic assignment in the course of a student’s education. Freshman may require more guidance with respect to collaboration, critical thinking, and research skills, whereas more advanced students may have developed these skills in earlier stages of their education. Foundational exercises about such topics should be required for the assignment depending upon the level of the student.

The final infographic assignment is available at https://scholarworks.montana.edu/xmlui/handle/1/14090. There are a number of ways that the infographic assignment can be evaluated. Sample rubrics, a final infographic submitted by a group in FRINQ, and comments about evaluating the infographic are also provided.

Future research should explore the infographic assignment at different times during a student’s education to parse apart the basic skills (e.g., finding resources, teamwork) that need to be taught at different levels. Likewise, the assignment could occur later in a course, after basic skills are taught. Students are not innately familiar with the intricacies of all digital technologies. Therefore, it is important to understand the degree to which students understand infographic creation and dissemination technologies and not to mistakenly assume that students are digital natives (16, 17).

## Conclusion: Infographics Pedagogy in Practice

The purpose of this study was to explore the degree to which an infographic assignment facilitated student learning around health sciences issues as well as the ways in which the assignment functioned as an effective teaching tool.

Requiring a commitment to be both learner and teacher, the integration of technology creates an educational environment that encourages participants to engage others. If a technology is new to the teacher, it may or may not be new to the student. As such, the infographic assignment for health sciences underwent two major revisions in piloting and then described in the current manuscript based on a participative process involving teachers and students. Teachers should show students what they already know, share perspectives on the values of a technology and an assignment, and ask students how they might (or already do) use the technology. Teaching is not about supplying answers to students, but about working with students “to create ‘active lifelong learners’ with ‘critical thinking skills’ and an ability to ‘think outside the box’” to pose, answer, and ask increasingly complex questions (18).

## Ethics Statement

This study was carried out in accordance with the recommendations of Portland State University Human Subjects Research Review Committee (HSRRC) with written informed consent from all subjects. All subjects gave written informed consent in accordance with the Declaration of Helsinki. The protocol was approved by the Portland State University Human Subjects Research Review Committee (HSRRC).

## Author Contributions

JDS initially conceived and designed the research and infographic assignment. BI, CS, AM, and CBS provided input regarding research design and infographic assignment development. JDS led manuscript composition and BI, CS, AM, and CBS contributed to drafting and revising of the manuscript. JDS and CBS collected pilot data. BI, CS, and AM collected study data. All authors analyzed and interpreted data.

## Conflict of Interest Statement

The authors declare that the research was conducted in the absence of any commercial or financial relationships that could be construed as a potential conflict of interest.
