# Corrigendum: Teaching Undergraduate Students to Visualize and Communicate Public Health Data with Infographics

**DOI:** 10.3389/fpubh.2017.00363

**Published:** 2018-01-17

**Authors:** Justin D. Shanks, Betty Izumi, Christina Sun, Allea Martin, Carmen Byker Shanks

**Affiliations:** ^1^MSU Library, Montana State University, Bozeman, MT, United States; ^2^OHSU-PSU School of Public Health, Portland State University, Portland, OR, United States; ^3^Community Health Program, Portland State University, Portland, OR, United States; ^4^Food and Health Lab, Montana State University, Bozeman, MT, United States

**Keywords:** infographics, health education, pedagogy, undergraduate education, communicating science

In the original article, there was an error. There is no Appendix. A correction has been made to the Discussion: Recommendations for Assignment Framework, paragraph two.

The final infographic assignment is available at https://scholarworks.montana.edu/xmlui/handle/1/14090.

The authors apologize for this error and state that this does not change the scientific conclusions of the article in any way.

The original article has been updated.

## Conflict of Interest Statement

The authors declare that the research was conducted in the absence of any commercial or financial relationships that could be construed as a potential conflict of interest.

